# *CDH1* germline variants are enriched in patients with colorectal cancer, gastric cancer, and breast cancer

**DOI:** 10.1038/s41416-021-01673-7

**Published:** 2021-12-23

**Authors:** Elio Adib, Talal El Zarif, Amin H. Nassar, Elie W. Akl, Sarah Abou Alaiwi, Tarek H. Mouhieddine, Edward D. Esplin, Kathryn Hatchell, Sarah M. Nielsen, Huma Q. Rana, Toni K. Choueiri, David J. Kwiatkowski, Guru Sonpavde

**Affiliations:** 1 Department of Medicine, Brigham and Women’s Hospital, Harvard Medical School, Boston, MA USA; 2 Lank Center for Genitourinary Oncology, Dana-Farber Cancer Institute, Boston, MA USA; 3 Division of Hematology and Medical Oncology, Tisch Cancer Institute, Icahn School of Medicine at Mount Sinai, New York, NY USA; 4 Invitae Corporation, San Francisco, CA USA; 5 Division of Population Sciences, Center for Cancer Genetics and Prevention, Dana-Farber Cancer Institute, Boston, MA USA

**Keywords:** Cancer genomics, Genetics research

## Abstract

**Background and aims:**

*CDH1* germline variants have been linked to heritability in diffuse gastric (DGC) and lobular breast cancer (LBC). Studies have not yet assessed whether *CDH1* is a cancer-susceptibility gene in other cancers. Herein, we mapped the landscape of pathogenic and likely pathogenic (P/LP) germline variants in *CDH1* across various cancers and ethnicities.

**Methods:**

We evaluated *CDH1* germline P/LP variants in 212,944 patients at one CLIA-certified laboratory (Invitae) and described their frequency in 7 cancer types. We screened for *CDH1* variant enrichment in each cancer relative to a cancer-free population from The Genome Aggregation Database version 3 (gnomADv3).

**Results:**

*CDH1* P/LP variants were identified in 141 patients, most commonly in patients with DGC (27/408, 6.6%) followed by colorectal signet-ring cell cancer (CSRCC; 3/79, 3.8%), gastric cancer (56/2756, 2%), and LBC (22/6809, 0.3%). *CDH1* P/LP variants were enriched in specific ethnic populations with breast cancer, gastric cancer, CRC, LBC, DGC, and CSRCC compared to matched ethnicities from gnomADv3.

**Conclusion:**

We report for the first time the prevalence of P/LP *CDH1* variants across several cancers and show significant enrichment in *CDH1* P/LP variants for patients with CSRCC, DGC, and LBC across various ethnicities. Future prospective studies are warranted to validate these findings.

## Introduction

Germline variants in *CDH1*, which codes for the cell–cell adhesion protein E-cadherin, were first identified in families with hereditary diffuse gastric cancer (HDGC) [1]. Subsequent reports noted that individuals with germline *CDH1* pathogenic variants were predisposed to both DGC and lobular breast cancer (LBC) [2]. Most recently, Massari et al. reported that 7% of all *CDH1* mutations are present in non-gastric tumours with most being identified in patients with breast cancer [3]. Moreover, *CDH1* mutations are more likely to be detected in areas with a low incidence of gastric cancer [4]. However, most reports have focused on highly penetrant HDGC families, in which the cumulative risk of HDGC for *CDH1* mutation carriers is 70% by age 80 years for men and 56% for women, while the cumulative risk of LBC for women is estimated to be 42% by age 80 years [5]. A recent study found that among patients not exclusively ascertained based on strict HDGC criteria, the cumulative incidence of gastric cancer for individuals with pathogenic *CDH1* variants is significantly lower (42% at age 80 years) than what has been previously reported [6, 7].

Although there is no evidence that the risk of other cancer types in individuals with a *CDH1* variant is significantly increased [8], multiple case reports have noted the occurrence of CRC and appendiceal Signet-Ring Cell Carcinomas in *CDH1* variant carriers [5]. In one study, colon cancer was reported in 3 of 238 (1%) *CDH1* pathogenic variant carriers, with 1 case of SRCC. Most recently, Benesch et al. postulated through clinical observation and data from SEER that SRCC might be enriched among *CDH1* variant carriers with signet ring cell gastric cancer [9]. Notably, there was no increased risk of colon cancer in *CDH1* carriers compared to that of the SEER population [6].

The prevalence of *CDH1* pathogenic variants in patients with gastric cancer and other cancer types is unknown. Here we examine the prevalence of *CDH1* variants among 212,944 patients referred for genetic testing. We also examine germline cancer-risk variants by ethnicity across several tumour subtypes and identify, for the first time, *CDH1* variant enrichment in individuals with CRC and colorectal signet-ring cell cancer (CSRCC).

## Methods

### Patient cohort

Personal history information for 212,944 independent probands with cancer was obtained from submitted requisition forms and medical records. The cohort included patients with breast, CRC, gastric, head and neck, ovarian, pancreatic, and prostate cancer types. All patients completed clinical germline genetic testing at Invitae (San Francisco, CA) between 09/2014 and 06/2020. Patient data were de-identified before analysis and the Western Institutional Review Board provided study oversight and approval. Western Institutional Review Board protocol number 1167406 waived the requirement to obtain written patient informed consent.

### Germline genetic testing

The genes selected for sequencing for each patient were chosen by the ordering clinician; all the patients reported here had been chosen for *CDH1* analysis. Genomic DNA was extracted from whole blood using a QiaSymphony (Qiagen, Hilden, Germany). Targeted genes including *CDH1* were captured using Agilent (Santa Clara, CA) SureSelect probes or Integrated DNA Technologies (Coral, IL) xGen Lockdown probes at positions where SureSelect yield was inadequate. Clinically important regions of *CDH1* including all the coding exons and 10 to 20 base pairs of adjacent intronic sequences on either side of the coding exons were covered. Next-generation sequencing [10] was performed on the Illumina (San Diego, CA) MiSeq or HiSeq 2500 to at least 350× average coverage of 2 × 150 reads, with a minimum of 50× required at every targeted position. Stringent process controls were used to minimise read-depth variability, and up to eight anonymous blood samples were used as control specimens in each run to measure remaining coverage variability [11].

### Germline variant calling and assessment

Small indels and single-nucleotide variants were analysed using the Genome Analysis Toolkit [12]. Copy-number variant calls were performed using CNVitae [11]. Large structural variants were detected using split-read analysis. Candidate *CDH1* variants were classified as pathogenic or likely pathogenic (P/LP) if: they affected the structure of *CDH1*; conferred a truncating, initiation codon or splice donor/acceptor effect; if functional data showed an impact on protein function; or if pathogenicity was otherwise reported in the published literature [13]. Orthogonal technology was used to validate P/LP variants via Sanger sequencing or Multiplex Ligation-Dependent Probe Amplification [14]. Confirmed variants were interrogated using a refined American College of Medical Genetics and Genomics criteria (Sherloc) [15]. For each of the examined tumour subtypes, the frequency of pathogenic germline variants in *CDH1* relative to the number of patients sequenced was calculated.

### Ethnicity and enrichment analysis

Ethnicity was provided by all patients at the time of test ordering and was grouped based on categories reported in population databases. The following ethnicities were considered in the analysis: Ashkenazi Jewish, Asian, Black/African American, White/Caucasian, and Hispanic. For each ethnicity, we calculated the frequency of pathogenic germline variants relative to the number of patients in which the gene was tested.

For every cancer subtype, we compared the frequency of pathogenic variants in *CDH1* to the frequency of gene variants in an independent population derived from The Genome Aggregation Database version 3 (gnomAD v3) [16]. Two independent methods were followed: (1) All *CDH1* variants in gnomAD were reviewed in ClinVar [17]. Variants in gnomAD deemed P/LP variants in ClinVar were retained. (2) Variants reported in gnomAD at a frequency >0.01% were excluded. Moreover, missense and synonymous variants in *CDH1* from both the Invitae and gnomAD cohorts were excluded and only loss-of-function (LOF) variants were retained. LOF variants included frameshift, splice site, and nonsense variants, variants in the initiator codon, and exonic deletions.

For both analyses, the frequency of germline variants in each of the ethnic populations (Ashkenazi Jewish, Asian, Black/African American, Hispanic, and White/Caucasian) in the Invitae cancer cohort were compared to the frequency of these gene variants across various ancestries derived from gnomAD v3. *CDH1* variants were considered enriched in a cancer subtype if they met both criteria: (1) they were significantly more likely to occur in a specific ethnic population with cancer when compared to the same ancestry in gnomAD and (2) the *p*-value was significant in both “LOF” and “ClinVar” analyses.

### Statistical analysis

Two-sided Fisher’s exact test was used to calculate the odds ratios, 95% confidence intervals (CIs), and *p*-values of all enrichment analyses. For the enrichment analysis, we applied Benjamini–Hochberg correction for the number of independent tests conducted (significant *q*-value cutoff of <0.05).

## Results

### Germline landscape of *CDH1* variants in the Invitae cohort

**Fig. 1:**
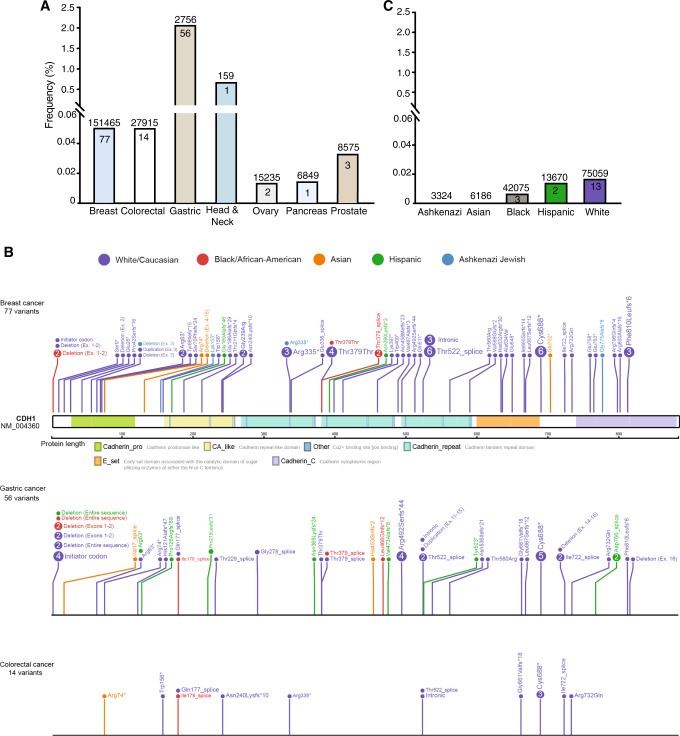
Frequency and landscape of germline *CDH1* variants. **a** Frequency of P/LP germline variants in *CDH1* in each of the seven cancer types. The numbers above each bar indicate the total number of patients tested for each cancer type; the number within each box indicates the number of P/LP variants. *Y* axis is frequency. **b** Landscape of P/LP germline variants in *CDH1* in 212,944 patients with cancer. The variants are labeled with carrier counts and coloured by their respective carriers’ ancestry (Caucasian: blue, African American: red, Asian: green, Ashkenazi Jewish: black, non-white Hispanic: orange) for breast cancer, gastric cancer, and colorectal cancer. **c** Frequency of P/LP germline variants in *CDH1* in each of the five ethnicities in the gnomAD v3 cohort. The numbers above each bar indicate the total number of subjects assessed by sequencing; the number within each box indicates the number of P/LP variants.

Of the 212,944 patients with cancer and available *CDH1* sequencing data, 151,465 had breast cancer (71.1%), 27,915 had CRC (13.1%), 15,225 had ovarian cancer (7.1%), and 18,339 (8.6%) had other cancer types (Fig. 1a and Table 1). Detailed clinical history was available on all 141 patients with P/LP variants in *CDH1* (Table S1.1). The most common cancer types in patients with P/LP *CDH1* variants were breast cancer (77 of 141, 54.6%), gastric cancer (56 of 141, 39.7%), and CRC (14 of 141, 9.9%). Among patients with breast cancer of known histology (*n* = 30), 8 (27%) had ductal and 22 (73%) had LBC. Notably, five probands with *CDH1* P/LP variants had concomitant gastric cancer and CRC. Variant type and location are shown in Fig. 1b and did not vary according to cancer type. The median age of onset of breast, colorectal, and gastric cancers among patients with *CDH1* P/LP variants was lower than that of the general population from the SEER cohort (Table S1.2) [18].

**Table 1:** Clinical and pathological characteristics of the 212,944 patients with cancer and available *CDH1* sequencing data.

	Patients with cancer	*N* = 212,944	Percent
	Gender		
	Female	188,416	88.5%
	Male	24,528	11.5%
Ethnicity			
	Ashkenazi	5836	2.7%
	Asian	8868	4.2%
	Black/African American	18,073	8.5%
	Hispanic	11,734	5.5%
	White	168,433	79.1%
Tumour Type			
	Breast cancer	151,465	71.1%
	Colorectal cancer	27,915	13.1%
	Gastric cancer	2756	1.3%
	Head and neck cancer	159	0.1%
	Ovarian cancer	15,225	7.1%
	Pancreatic cancer	6849	3.2%
	Prostate cancer	8575	4%

Among all major cancer types, gastric cancer had the highest frequency of P/LP variants (56 of 2756, 2%, 95% CI = 1.5–2.6%, Fig. 1a and Table S1.3) followed by head and neck (1 of 159, 0.6%, 95% CI = 0–3.5%) and breast cancer (77 of 151,465, 0.05%, 95% CI = 0.04–0.06%). Acknowledging that there were relatively small numbers of subjects in some categories (e.g. Ashkenazi Jewish with gastric cancer), none of the five ethnicities showed significantly higher *CDH1* P/LP variant frequency for any cancer type (*p*-values of pairwise comparisons in Table S1.4). Various population groups and their cohort sizes are shown in Supplementary Table S1.5.

### Enrichment analysis of major cancer subtypes

**Fig. 2:**
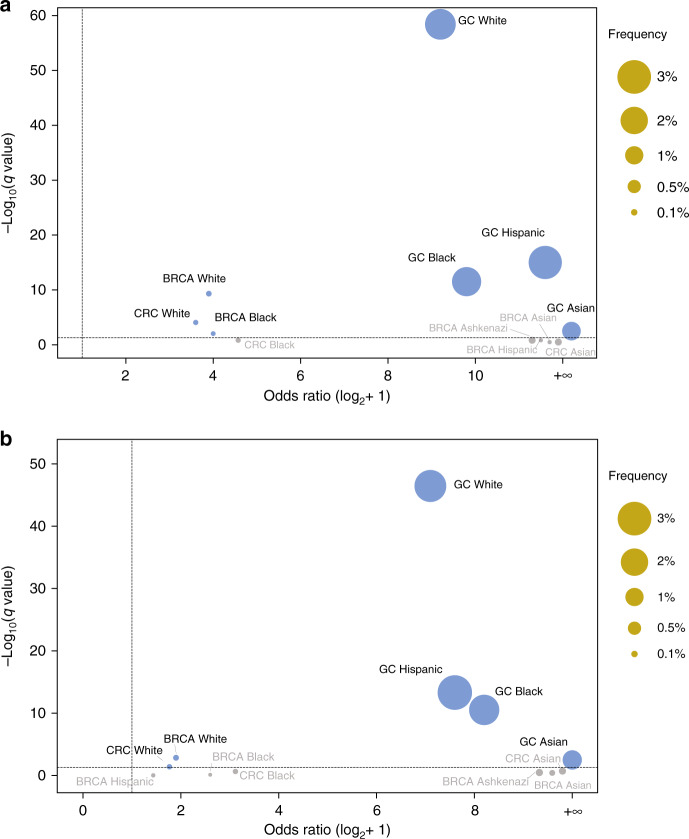
Enrichment analysis of *CDH1* variants. Enrichment of P/LP *CDH1* variants in patients with various cancer types using two independent methods (see Supplementary Methods). Fisher’s exact test was used to calculate the odds ratios (ORs) and 95% confidence intervals (CIs). A two-sided binominal test was used to compute the *p*-values. **a** Enrichment analysis including variants in gnomAD deemed P/LP in ClinVar. **b** Enrichment analysis including only loss-of-function variants in gnomAD and Invitae found at a frequency ≤0.01. Applying a false discovery rate of <0.05 (horizontal dotted line), germline variants in *CDH1* were enriched in Caucasians with CRC; African Americans/Blacks, Asians, Caucasians, and Hispanics with gastric cancer; and Caucasians with breast cancer when compared to corresponding ancestries from gnomAD v3. BRCA breast cancer, CRC colorectal cancer, GC gastric cancer.

We then conducted enrichment analysis (see methods) and found that the odds of P/LP germline variants in *CDH1* among African Americans, Asians, Caucasians, and Hispanics with gastric cancer in our cohort was significantly higher (126-fold to infinity) in comparison to the odds in the corresponding gnomAD ancestry cohorts (Figs. 1c and 2a, b and Table S1.5). Caucasians with breast cancer and CRC were enriched for *CDH1* germline variants compared to Caucasian controls from gnomAD. The odds ratio was also increased for *CDH1* variants in several other ethnicities for each of breast and colorectal cancer, though in general to a smaller degree, and false discovery rate-corrected *q*-values were not significant.

### Enrichment of *CDH1* variants in DGC and LBC

**Fig. 3:**
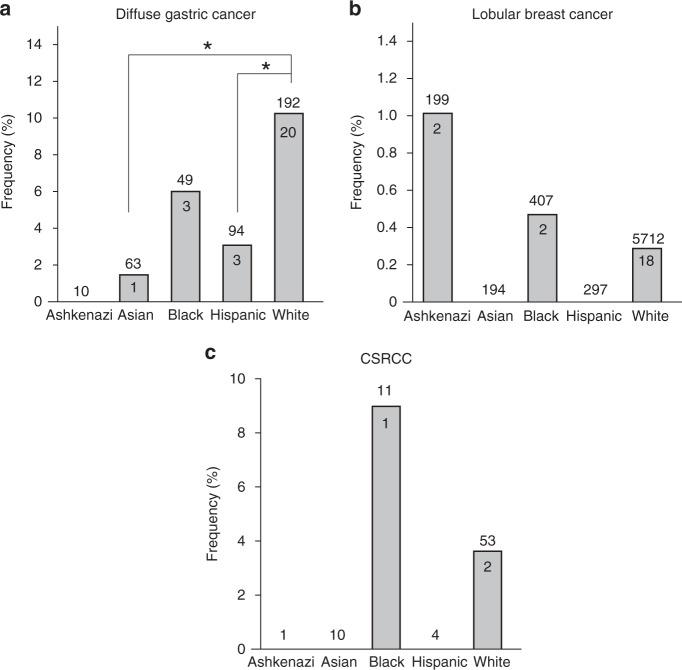
Ethnicity-specific enrichment of *CDH1* variants in three cancer subtypes. **a**–**c** Frequency of pathogenic and likely pathogenic germline variants in *CDH1* in each of the five ethnicities in the Invitae cohort for diffuse gastric cancer (**a**), lobular breast cancer (**b**), and CSRCC (**c**). A two-sided binomial test was used to compute the *p*-values. *p-value < 0.05.

We then analysed histology-specific associations and found germline P/LP variants in *CDH1* in 6.6% (27 of 409) of patients with DGC and 0.3% (22 of 6955) of patients with LBC. The median age at diagnosis of patients with DGC and LBC harbouring *CDH1* P/LP variants was 42 (range 19–75) and 48 years (range 41–66), respectively. Interestingly, among patients with DGC in our cohort, Caucasians harboured significantly more *CDH1* P/LP germline variants compared to Asians and Hispanics (20/192, 10.4% vs 1/63, 1.6%, *p* = 0.032 and 3/94, 3.2% vs 0/13670, 0%, *p* = 0.038 respectively, Fig. 3a and Table S1.6). However, in LBC, none of the five ethnicities showed significantly higher *CDH1* P/LP variant frequency (Fig. 3b and Table S1.6).

Enrichment analysis with gnomAD showed that African Americans and Caucasians with LBC and DGC were enriched for P/LP *CDH1* germline variants. Ashkenazi Jews, Asians, and Hispanics harboured significantly more *CDH1* P/LP variants compared to controls from gnomAD in LBC and DGC, respectively (Table S1.7).

### Prevalence of *CDH1* variants in CSRCC and enrichment compared to gnomAD

Prior case reports suggested that an association between CSRCC and *CDH1* germline carriers exists [5, 19]. We found that 3.8% of patients with CSRCC (3 of 79) harboured *CDH1* P/LP variants. Age at diagnosis for two of the three patients was known (35 and 41 years). Compared to corresponding controls from gnomAD, African Americans and Caucasians with CSRCC were enriched for P/LP *CDH1* germline variants (African American LOF analysis: *q* = 0.0017, OR = 1365, 95% CI = 24–16,384; African American ClinVar Analysis: *q* = 0.001, OR = 1365, 95% CI = 24–16,384; Caucasian LOF analysis: *q* = 0.0001, OR = 226, 95% CI = 24–996; Caucasian ClinVar Analysis: *q* < 0.0001, OR = 969, 95% CI = 80–8192; Fig. 2a, b and Table S1.7, see “Methods”). However, we consider these findings tentative given the small number of patients within each ethnic group in our study (Fig. 3c and Table S1.6).

## Discussion

The prevalence of *CDH1* germline variants in patients with various cancer types is still not well-described. Herein, we found *CDH1* germline carriers in about 7% of patients with DGC and 3.8% of patients with CSRCC. To date, there is conflicting evidence regarding the prevalence of *CDH1* P/LP germline variants in LBC. In our study, among 6809 patients with LBC, approximately 0.3% harboured germline variants in *CDH1*. This frequency is lower than what is reported in prior studies (1–8%), which focused mainly on patients with early-onset or bilateral disease [20, 21]. In a comprehensive review of hereditary LBC, Corso et al. emphasised the importance of surveillance in *CDH1* P/LP variant carriers [22]. With increasing knowledge about LBC risk factors, *CDH1* germline genetic testing in high-risk families remains paramount.

Prior studies have led to conflicting results regarding the prevalence of *CDH1* germline variants in Asians vs non-Asians. Despite the high incidence of gastric cancer in East Asian countries, previous work has suggested that low detection rates for germline *CDH1* variants are identified in Asians compared to countries with a lower incidence of gastric cancer [23]. More recently, a study [24] of 105 Japanese patients with DGC found that germline variants in *CDH1* occured in 14 patients (13.3%) and showed that Japanese patients with gastric cancer were four times more likely to harbour *CDH1* variants compared to TCGA (The Cancer Genome Atlas) non-Asian populations with gastric cancer. In our study, we found that the prevalence of *CDH1* P/LP variants is significantly higher in Caucasians with DGC (10.4%) compared to Asians (1.6%, *p* = 0.032) and Hispanics (3.2%, *p* = 0.038). One possible explanation for this difference is that non-Japanese Asians, which likely represent a sizable portion of our Asian population, may have lower levels of *CDH1* variants compared to Japanese Asians. Another observation is that all 14 variants identified by Suzuki et al. [24] are classified as benign, likely benign, or of uncertain significance according to Invitae guidelines and ClinVar reports, and hence would not be P/LP variants by our criteria. Whether those 14 variants are truly non-pathogenic or are unrecognised but significant variants in *CDH1* remains to be determined.

Histology-specific enrichment analysis validated prior associations between *CDH1* germline variants in LBC and DGC and identified a novel association with CSRCC [9]. CSRCC is an aggressive adenocarcinoma subtype with a poor prognosis overall and thus determining cancer-risk susceptibility genes presents an unmet need. Guidelines for genetic testing factor in the underlying likelihood of identifying a germline variant [25]. Thus, accurate estimates of germline prevalence, identified herein, may alter recommendations for different patient populations. Consideration should also be given to colonoscopy surveillance in *CDH1* carriers. Notably, prior work [6] did not identify an increased risk of developing CRC in *CDH1* carriers compared to patients from the SEER cohort but was underpowered to assess for associations in patients with CSRCC. In our cohort, *CDH1* variant enrichment was observed among Caucasian patients with CRC.

When comparing the prevalence of *CDH1* P/LP variants across the different ethnicities within each cancer type or subtype, none of the five ethnic populations showed significantly higher *CDH1* P/LP variant frequency, except for the aforementioned observation in DGC. Thus, the enrichment seen when comparing to gnomAD cohorts is independent of ethnicity but was not seen in some comparisons likely due to the lack of statistical power.

*CDH1* is a tumour suppressor, and the vast majority of variants detected here were LOF (128 of 141, 90.8%, 95% CI = 84.9–94.5%), and distributed throughout the coding sequence. A prior study reported significant enrichment of *CDH1* germline variants located in the PRE-PRO region (amino acid 1–115) in HDGC families affected by CRC. In addition, patients harbouring *CDH1* variants in the linker region (regions shown in white, Fig. 1B) were significantly less likely to develop breast cancer [26]. In this dataset, there was no association between the location of *CDH1* variant and the development of individual cancers.

Our study has several limitations. First, the selection of patients for genetic testing was influenced by clinical judgement and was likely skewed towards individuals with a significant suspicion for heritable pathogenic variants. Second, personal and family history data were obtained from genetic testing requisition forms and were not confirmed by direct review of the medical records or other data sources. Ethnicity information was provided by the subjects with no confirmation. Similarly, there was no central pathological confirmation of either the cancer type or subtype. We caution against overinterpreting ethnicity-specific *CDH1* associations as only a handful of carriers may drive enrichment in a small cancer cohort.

In the largest study to date evaluating *CDH1* germline variants, we found significant enrichment of P/LP variants in patients with CSRCC, CRC, breast, and gastric cancer. Importantly, we found that the frequency of P/LP *CDH1* variants in multiple cancer types did not vary according to ethnicity for the most part. This is the first report on the prevalence of *CDH1* variants in African American and Hispanic populations and indicates that these populations have the same frequency of P/LP variants as Caucasians and should be subject to the same germline testing and screening considerations.

## Supplementary information

Supplementary Table S1

Supplementary Table Legends

## Data Availability

Data will be available in Supplementary Table S1 and Supplementary Materials.
