# Supplementary material for: CDH1 germline variants are enriched in patients with colorectal cancer, gastric cancer, and breast cancer
Source: Br J Cancer. 2021 Dec 23;126(5):797–803. doi: 10.1038/s41416-021-01673-7 (PMC8888603; doi:10.1038/s41416-021-01673-7)
Supplement: Supplementary file 2 — Supplementary Table Legends [file 41416_2021_1673_MOESM2_ESM.docx]

**Supplementary Materials**

**Supplementary Table S1.** Clinical, genomic, and pathological characteristics of 212,944 cancer patients with *CDH1* testing.

**S1.1.** Master table showing the clinical, pathological, and genomic data for 141 cancer patients with pathogenic or likely pathogenic *CDH1* variants.

**S1.2.** Median age of onset of gastric, breast, and colorectal cancers in patients with *CDH1* germline variants stratified by gender and compared to the general population.

**S1.3.** Frequency of *CDH1* germline variants in each cancer type and subtype.

**S1.4.** Enrichment analysis for *CDH1* comparisons between various ancestral populations in each of the cancer types. *p*-values are shown, Fisher exact test.

**S1.5.** Enrichment analysis comparing the frequency of germline variants in *CDH1* between the Invitae cancer types and the gnomAD v3 population. *p*-values were adjusted for multiple comparisons and a cut-off q value <0.05 was considered significant. Two independent enrichment analyses were performed, one using ClinVar interpretation and the other using LOF variants (see supplementary methods).

**S1.6.** Enrichment analysis for *CDH1* comparisons between various ancestral populations in each of the cancer subtypes. *p*-values are shown, Fisher exact test.

**S1.7.** Enrichment analysis comparing the frequency of germline variants in *CDH1* between diffuse gastric, lobular breast, signet-cell colorectal cancer and the gnomADv3 population. *p*-values were adjusted for multiple comparisons and a cut-off q value <0.05 was considered significant. Two independent enrichment analyses were performed (see supplementary methods).
